# Cumulative kinetics of epigenetic abnormalities during initiation and progression of Adult T-cell Leukemia/Lymphoma (ATLL)

**DOI:** 10.1186/1742-4690-8-S1-A195

**Published:** 2011-06-06

**Authors:** Takashi Oka, Hiaki Sato, Lamia A Al-Kader, Yoko Shinnou, Kana Washio, Katsuyoshi Takata, Ichiro Murakami, Atae Utsunomiya, Mamoru Ouchida, Kiyoshi Takahashi, Tadashi Yoshino

**Affiliations:** 1 Department of Pathology, Graduate School of Medicine, Dentistry and Pharmaceutical Sciences, Okayama University, Okayama, 700-8558, Japan; 2 Department of Medical Technology, Graduate School of Health Science, Okayama University Medical School, Okayama, 700-8558, Japan; 3 Department of Molecular Pathology, Tottori University Medical School, Yonago, Tottori, 683-8503, Japan; 4 Department of Hematology, Imamura Bun-in Hospital, Kagoshima, 890-0064, Japan; 5 Department of Molecular Genetics, Graduate School of Medicine, Dentistry and Pharmaceutical Sciences, Okayama University, Okayama, 700-8558, Japan

## 

HTLV-1 causes ATLL in 3-5% of infected individuals after a long latent period of 40-60 years. ATLL is divided into four stages: namely, smoldering, chronic, lymphoma and acute types. The smoldering and chronic types are indolent, but the acute and lymphoma types are aggressive ATLL characterized by resistance to chemotherapy and a poor prognosis. Such a long latent period suggests that a multi-step leukemogenic/lymphomagenic mechanism is involved in the development of ATLL, although the critical events in the progression have not been characterized. To determine whether epigenetic abnormalities are playing important roles in the progression of ATLL, we analyzed the methylation profiles, showing that number of CpG island methylated genes increased with disease progression and aberrant hyper-methylation in specific genes was detected even in HTLV-1 carriers and correlated with progression to ATLL. The CpG island methylator phenotype (CIMP) was observed most frequently in lymphoma type ATLL and was also closely associated with the progression and crisis of ATLL. The high number of methylated genes and increase of CIMP incidence were shown to be unfavorable prognostic factors and correlated with a shorter overall survival with the Kaplan-Meyer analysis. Increase of aberrant DNA methylation density was observed during the progression of an ATLL patient. The present findings strongly suggest that the multi-step accumulation of aberrant CpG methylation in specific target genes and the presence of CIMP are deeply involved in the initiation and progression of ATLL not only epidemiologically but also in the clinical course of a specific ATLL patient.

